# Urinary extracellular vesicles in childhood kidney diseases

**DOI:** 10.1007/s00467-023-06243-y

**Published:** 2023-12-13

**Authors:** Yutaka Harita

**Affiliations:** Department of Pediatrics, The University of Tokyo Hospital, 7-3-1 Hongo, Bunkyo-Ku, Tokyo, 113-8655 Japan

**Keywords:** Urinary extracellular vesicles, Exosome, Liquid biopsy, Chronic kidney disease, ELISA

## Abstract

Most biological fluids contain extracellular vesicles (EVs). EVs are surrounded by a lipid bilayer and contain biological macromolecules such as proteins, lipids, RNA, and DNA. They lack a functioning nucleus and are incapable of replicating. The physiological characteristics and molecular composition of EVs in body fluids provide valuable information about the status of originating cells. Consequently, they could be effectively utilized for diagnostic and prognostic applications. Urine contains a heterogeneous population of EVs. To date, these urinary extracellular vesicles (uEVs) have been ignored in the standard urinalysis. In recent years, knowledge has accumulated on how uEVs should be separated and analyzed. It has become clear how uEVs reflect the expression of each molecule in cells in nephron segments and how they are altered in disease states such as glomerular/tubular disorders, rare congenital diseases, acute kidney injury (AKI), and chronic kidney disease (CKD). Significant promise exists for the molecular expression signature of uEVs detected by simple techniques such as enzyme-linked immunosorbent assay (ELISA), making them more applicable in clinical settings. This review presents the current understanding regarding uEVs, emphasizing the potential for non-invasive diagnostics, especially for childhood kidney diseases.

## Introduction

Most biological fluids, including urine, plasma, serum, saliva, seminal fluid, amniotic fluid, and breast milk, contain extracellular vesicles (EVs) [1, 2]. EVs contain DNA, RNA, lipids and proteins, and are delimited by a lipid bilayer. They do not contain a functional nucleus and cannot replicate [3]. The physiological and molecular characteristics of EVs have the potential to provide valuable information into the cellular origin and pathophysiological condition. Consequently, they could be effectively utilized for diagnostic and prognostic applications [4]. Historically, their presence has been confirmed by electron microscopy since the 1960s [5]. Since then, there has been exponential growth in the research performed to characterize EVs and use them across various medical fields.

EVs are abundant in urine [2, 4]. However, they have been ignored in the standard urinalysis. Recent investigations have developed various purification and analysis methods, demonstrating that using urinary extracellular vesicles (uEVs) to diagnose and treat kidney diseases holds great promise. In 2019, *Pediatric Nephrology* released a landmark review that compiled a wealth of information on the subject [6]. Subsequently, significant progress has been made in the field of research relevant to EVs or uEVs. This review presents the current advances on uEVs, emphasizing the potential for non-invasive diagnostics, especially for childhood kidney diseases.

## Variety of EVs in urine

EVs are a heterogeneous population that can vary in size, shape, composition, biogenic mechanisms, and specific biomarkers [3]. Urinary EV samples often contain a heterogeneous mixture of EVs, including exosomes, ectosomes (more commonly called microvesicles and microparticles), arrestin domain-containing protein 1-mediated microvesicles (ARMM), apoptotic bodies, autophagic extracellular vesicles, or even non-vesicular compartments such as exomeres [7].

Although the classification of EVs is continually evolving, our current knowledge of their biogenesis has broadly categorized classical EVs into two main types: exosomes and ectosomes/microvesicles [6–8].

1. Exosome
  - Origin: Intraluminal vesicles (ILVs) are released into the extracellular space as exosomes when endosomes known as a multivesicular bodies (MVB) carrying ILVs fuse with the plasma membrane [8].
  - Size: Exosomes are small with a size range of 40–150 nm.
  - Composition: Exosomes contain a diverse cargo of molecules, including proteins, lipids, RNAs (such as messenger RNAs, microRNAs, and long non-coding RNAs), and other signaling molecules. Classical exosomes do not natively contain dsDNA and do not associate with other particles or proteins that contain dsDNA [7].
  - Surface markers: classic tetraspanins such as CD63, CD81, and CD9.
2. Ectosome/microvesicle
  - Origin: Ectosomes/microvesicles arise from outward protrusions of plasma membrane. They are generated when they are excised and shed into the extracellular space. Membrane protrusions such as filopodia, cilia, and microvilli can shed small ectosomes.
  - Size: Ectosomes range in diameter from less than 100 nm to several micrometers and contain a variety of vesicle types, including microvesicles (typically 0.2–1 nm in diameter) and huge oncosomes (> 1 μm) [8].
  - Composition: They contain a diverse range of molecules, including membrane proteins, lipids, RNAs, cytosolic proteins, and other signaling molecules. They may enclose free cytosolic DNA.
  - Surface markers: annexin A1 (ANXA1), tumor susceptibility gene 101 protein (TSG101), and arrestin-domain-containing protein 1 (ARRDC1) [7].

Essentially, exosomes and ectosomes/microvesicles differ in their release mechanisms. However, it is not practical to capture the moment of release of each vesicle by live imaging to distinguish between them. Therefore, consensus on the classification based on a particular biogenesis pathway has yet to emerge. Note that although major EV cargo sorting mechanisms such as the endosomal sorting complexes required for transport (ESCRT) machinery or classic tetraspanins have been linked to exosomes; similar biogenesis machinery might be engaged at different locations in the cell to create ectosomes/microvesicles. Therefore, the presence or absence of any particular tetraspanin in a mixture of EVs cannot be used to determine whether the EVs are exosomes or ectosomes [8].

As mentioned above, the terms “exosome” and “ectosome/microvesicle” have historically been burdened by multiple contradictory definitions and inaccurate expectations regarding their unique biogenesis [1]. The recent guideline by the *Journal of Extracellular Vesicles* recommended that authors utilize operational terms for different subtypes of EVs. These terms should be based on specific physical characteristics, such as size (referred to as “small EVs” (sEVs) and “medium/large EVs” (m/lEVs), with defined size ranges, for example, < 100 nm or < 200 nm for small EVs, and > 200 nm for medium/large EVs), or density (low, middle, high, with each range precisely defined) [1].

## Physical and molecular characterization of uEVs

The discovery of EVs in urine [9] opened a new, fast-growing scientific field [10]. Previous studies have conducted detailed characterizations of the morphological or molecular properties of vesicles present in entire urine or uEVs [11–16]. It is important to note that these properties vary depending on their purification [10] or characterizing methods [14]. In order to optimize and standardize uEVs research, the Urine Task Force of the Rigor and Standardization Subcommittee of ISEV, consisting of nephrologists, urologists, cardiologists, and biologists, published a position paper in 2021 [10], which summarizes the recent method to separate and characterize uEVs and present challenges and gaps in current analyses.

uEVs contain vesicles of various sizes, but most are in the 30 to 200 nm range [14, 17]. We investigated the quantity and size distribution of uEVs derived from children using nanoparticle tracking analysis (NTA). The average number of uEVs obtained from healthy control samples was determined to be about 20 × 10^9^ particles/mL. The mean size ± *SD* of uEVs was 137.9 ± 2.5 nm, peaking at 117.4 ± 1.0 nm [18].

The size and shape of uEVs can undergo modifications in a pathological condition. In the previous study, we found no difference in the average size of uEVs from children diagnosed with chronic kidney disease (CKD) and from the control. Nonetheless, it was observed that the comparative density of peak size exhibited a notable decrease among patients with CKD in comparison to the control, implying a deviation from the typical relatively homogeneous population of vesicles [18]. It should also be noted that the size of uEVs varies with dilution [14].

Proteomic analysis indicates that a significant majority, notably 99.96%, of the proteins identified in urinary extracellular vesicles exhibit distinct characteristics that are commonly associated with cells originating from the urogenital tract [4], and the most prevalent sources of these vesicles are glomerular, tubular, prostate, and bladder cells [4]. The protein composition of uEVs from healthy children contains several thousands of molecules, including several common markers for different EV subpopulations (exosome (CD63 and CD9), classical microvesicles (ANXA1), and ARMM (TSG101)). Pathway analysis demonstrated that many pathways are enriched, including lysosomes, metabolic pathways, endocytosis, and proximal tubule transport [18]. The proteome data suggests that uEVs are released from several nephron segments [18].

In addition to proteins or lipids, uEVs contain RNAs. Among the classes of RNAs, microRNA (miRNA), consisting of roughly 22 nucleotides, has attracted significant attention. RNAs (mRNAs and miRNAs) in uEVs have been comprehensively reviewed in previous influential publications [4, 19–21].

## uEVs as biomarkers for kidney diseases

There is a growing body of research on the alterations of molecular compositions in uEVs in many renal and urological disorders [4, 6, 22–24].

### Glomerular disorders

Nephrotic syndrome is the most studied pediatric disease for uEVs. Santorelli et al. analyzed uEV protein profiles of children with steroid-sensitive, steroid-dependent, and steroid-resistant nephrotic syndrome. They demonstrated that uEV protein profiles could differentiate steroid-resistant patients from other idiopathic nephrotic syndrome patients and controls [25]. As a protein biomarker in uEVs, Zhou et al. reported Wilms’ tumor 1 (WT1) transcription factor, which is widely recognized as a marker for differentiated podocytes, as a potential biomarker for early progression and treatment-induced regression of podocyte injury in childhood focal segmental glomerulosclerosis (FSGS) or steroid-sensitive nephrotic syndrome [26]. However, the other study questions its importance as a biomarker in childhood nephrotic syndrome [27]. Recently, podocyte-derived large EVs (annexin V- and podoplanin-positive) have been reported to be increased in pediatric idiopathic nephrotic syndrome [28]. A unique miRNA profile in uEVs has also been reported. A study analyzing adult patients found that miR-1915 and miR-663 were downregulated in patients with FSGS compared to minimal change disease and controls, whereas the urinary levels of miR-155 were upregulated in patients with FSGS [29]. Huang et al. analyzed pediatric patients and demonstrated that miR-193a is a potential biomarker for primary FSGS [30]. By comparing miRNAs in uEVs from children with nephrotic syndrome and controls, Chen et al. found that miR-194-5p, miR-146b-5p, miR-378a-3p, miR-23b-3p, and miR-30a-5p were increased in patients, and some of them were positively correlated with the urine protein content [31].

For IgA nephropathy, Moon et al. demonstrated four proteins (aminopeptidase N, vasorin precursor, α-1-antitrypsin, and ceruloplasmin) as protein biomarkers to differentiate IgA nephropathy from thin basement membrane nephropathy [32]. More recently, Feng et al. found that chemokine (C–C motif) ligand 2 (CCL2) mRNA was upregulated in uEVs from patients with IgA nephropathy [33]. Exosomal CCL2 exhibited a significant correlation with tubulointerstitial inflammation and C3 deposition, and elevated levels of CCL2 mRNA at the timing of kidney biopsy were linked with the later decline in kidney function. Min et al. analyzed miRNAs in adult patients and demonstrated that miR-29c, miR-146a, and miR-205 may potentially serve as biomarkers for IgA nephropathy [34].

### Tubular disorders

uEVs mirror the expression levels of renal tubular transporters in the kidney [35], making them applicable as a source of biomarkers for a wide range of renal tubular disorders. For instance, the uEVs derived from individuals diagnosed with Bartter syndrome type 1 and Gitelman syndrome have diminished or non-existent Na–K–2Cl cotransporter (NKCC2) and Na–Cl cotransporter (NCC) expression levels, reflecting its expression level in renal tissue [36, 37]. In contrast, a compensatory upregulation of Na^+^/H^+^ exchanger 3 (NHE3), β-subunit of the epithelial Na^+^ channel (β-ENaC), and pendrin was observed in uEVs and kidney biopsies obtained from individuals diagnosed with Gitelman syndrome [36]. It is also known that such pathology-related changes in molecular expression in the tubules can be detected by uEVs in cystinosis [38], primary aldosteronism [39, 40], Cushing syndrome with hypertension [41], pre-eclampsia [42], and renal tubular acidosis [43].

### Other rare congenital kidney diseases

ADPKD is among the most studied rare diseases concerning its uEVs [44]. In uEVs from affected individuals, levels of polycystin-1 (PC1) and polycystin-2 (PC2) were significantly reduced, and transmembrane protein 2 (TMEM2), a protein with homology to fibrocystin, was upregulated. The PC1/TMEM2 ratio correlated inversely with height-adjusted total kidney volume [45]. In other reports, periplakin, envoplakin, villin-1, and complement C3 and C9 [46] or CD133 [47] were found to be overexpressed in uEVs in adult ADPKD patients, suggesting their possible role in the pathogenesis. Regarding miRNA, Magayr et al. found that miR-192-5p, miR-194-5p, miR-30a-5p, miR-30d-5p, and miR-30e-5p were downregulated in patients and were correlated with baseline eGFR and mean kidney length [48]. Noticeably, they confirmed altered expression of these microRNAs in a validation set of cystic kidney tissues at its early stage, suggesting that miRNA in uEVs reflect changes in renal tissue.

Medullary sponge kidney disease (MSK) is characterized by malformation of the terminal collecting ducts in the renal pyramids that results in nephrocalcinosis and recurrent kidney stones. The most significant biomarker of uEVs in MSK was laminin subunit α2, a major component of the basement membrane [49], which is thought to promote cyst formation. In the other analysis conducted on patients with MSK and patients with idiopathic calcium nephrolithiasis as controls, Ficolin 1 and Complement component 4-binding protein were found to be upregulated while Mannan-binding lectin serine protease 2 was downregulated in MSK [50]. This study revealed that the lectin pathway may have a role in the abnormal polarization of the cells and the cystogenesis in MSK.

In uEVs from patients with autosomal dominant tubulointerstitial kidney disease associated with the *MUC1* gene (ADTKD-MUC1), proteins that are functionally linked to vesicular transport and membrane dynamics, such as vacuolar protein sorting-associated protein (VTA1), were significantly reduced [51].

As there are numerous unknowns surrounding the origins and progression of these rare diseases, the disease-specific signatures of uEVs will help us to identify unrecognized key biological components involved in their pathogenesis [44].

### Acute kidney injury

Using animal models of acute kidney injury (AKI), activating transcription factor 3 (ATF3) [52, 53] and Fetuin A [54] were identified as candidate protein biomarkers in uEVs, and they were also confirmed in human adult AKI. Interestingly, RNA levels of ATF3 in uEVs are also increased in a mouse AKI model and human AKI [55]. Likewise, NHE3 in urine membrane fraction was identified as a marker for critically ill patients with AKI [56]. Recently, this study was validated and NHE3 in uEVs was found to be a useful biomarker in various animal AKI models and sepsis-induced AKI [57].

### Chronic kidney disease

Childhood CKD has a significant impact on morbidity and mortality and its association with many medical complications that extend beyond the pediatric age group. To date, plenty of research investigations have been undertaken to search for urinary (non-uEVs) biomarker candidates for CKD [58, 59]. These biomarkers reflect pathophysiologic processes of tubulointerstitial injury, inflammation, repair, and fibrosis [58].

There have been several reports on uEV biomarkers in adult CKD, without imposing restrictions on the underlying diseases [60–62]. In contrast to adult CKD, the primary causes of childhood CKD are congenital or genetic diseases characterized by a quantitative and qualitative decline in the number of functioning nephrons. The primary etiology is typically attributed to congenital abnormalities of the kidney or urinary tract (CAKUT) [63, 64]. CAKUT encompasses a diverse spectrum of congenital illnesses, such as kidney agenesis, hypo/dysplastic kidney, posterior urethral valves, and congenital obstructive uropathy. The diagnosis of CAKUT currently relies heavily on imaging studies such as ultrasound. The progression of childhood CKD has distinct characteristics compared to its adult counterpart, primarily characterized by less systemic and inflammatory manifestations [65]. Patients may not present alterations in serum creatinine levels due to compensatory hypertrophy of residual nephrons. They frequently do not present with hematuria, and creatinine and proteinuria increase relatively late during kidney damage [66, 67]. Childhood CKD can progress to kidney failure, from the early postnatal period to late adulthood [68]. Due to the distinct differences in the etiology and pathophysiology of CKD, there is a pressing need for targeted research in children.

We hypothesized that there may be common changes in uEVs when functional nephrons are reduced. In order to investigate the presence of differentially expressed proteins in uEVs derived from individuals diagnosed with bilateral kidney hypoplasia, a condition characterized by a congenital reduction in the number of nephrons, we conducted a quantitative proteomic analysis [18]. A total of 135 proteins exhibiting discriminatory characteristics were identified. One of the molecules that exhibited a decrease in uEVs in hypodysplasia was mucin1 (MUC1), whose expression is restricted to the distal tubule and collecting duct. MUC1 fulfills diverse roles in physiological and pathological states [69–71]. Another example of EV signature was proximal tubule-specific maltase-glucoamylase (MGAM), which was increased in uEVs in renal hypodysplasia. The uEV expression patterns of 135 molecules reflected decreased kidney function by not only renal hypoplasia, but also other forms of CAKUT or ciliopathies [18]. These data suggested that the uEV signature may reflect decreased kidney function in childhood CKD.

## Application of uEVs as a novel urine test

The application of uEVs to diagnostics of kidney and urogenital diseases is accelerating. Recently, the Food and Drug Administration (FDA) approved the initial uEV-based assay (ExoDx) in urology, which utilizes an RNA signature as a non-invasive screening technique for prostate cancer [72, 73]. The ExoDx Prostate assay has been shown to provide individualized risk assessment of clinically significant prostate cancer at initial biopsy [74, 75]. In this system, urine samples can be collected at home, shipped to a central laboratory, and stored. RNAs in uEVs are extracted, and reverse transcriptase polymerase chain reaction (RT-qPCR) is performed. Despite its high cost, ExoDx may help avoid unnecessary biopsies.

We have been exploring the potential for utilizing the outcomes of quantitative proteome analysis of uEVs straightforwardly, practically, and cost-effectively. We developed an ELISA that quantifies substances on the surface of uEVs [76]. With the aid of this straightforward ELISA, we verified that MUC1 expression was reduced in children with CKD and that it helps to identify individuals with impaired kidney function [18]. As ELISA can be performed in a conventional clinical laboratory, the protein content of uEVs can be a robust biomarker candidate. This is especially true when screening for conditions for which standard urinalysis is not able to detect abnormalities.

## Promises and caveats of uEVs as a liquid biopsy of the kidney

As mentioned above, contents of uEVs can be affected by the physiological and pathophysiological renal conditions [4, 77]. However, whether uEVs faithfully reflect molecular changes in the kidney has been debated. In this regard, Wu et al. performed quantitative proteomics analyzing approximately 1000 proteins identified in uEVs and corresponding kidney tissue [78]. They demonstrated significant associations between the protein amounts in uEVs and those in whole kidneys. Noticeably, transmembrane proteins exhibited higher positive correlations compared to cytoplasmic proteins. Moreover, changes in protein expression levels in the kidney tissues can be detected as alteration in the composition of uEVs. These data offer compelling evidence to support the utilization of uEVs as reliable indicators of kidney disease.

Another crucial aspect to be considered related to the potential factors that may influence the content of uEVs is their excretion rate. As the cells in the kidney are the primary source of uEVs, nephron mass should determine uEV excretion. Blijdorp and colleagues [79] established a significant association between uEV excretion rate and renal parameters, including total kidney volume, estimated glomerular filtration rate, and creatinine clearance. By examining urine samples obtained from individuals who underwent donor nephrectomy, the researchers established that the excretion of compensatory uEV due to hypertrophy primarily took place in the proximal tubule. The rat models (uninephrectomy and 5/6 nephrectomy) have also confirmed that the extent of hypertrophy corresponds to the uEV excretion rate [79]. Hence, to facilitate the comparison of urine samples from several individuals or different time points within the same individual, it is imperative to consider the excretion rate and variability of uEVs, which are influenced by functional nephron mass and compensatory hypertrophy.

Furthermore, it should be noted that except for several articles [18, 25–28, 30, 31], most of the studies cited in this review analyzed uEVs from adult patients with kidney diseases. These data cannot be simply applied to childhood diseases due to various factors such as differences in urine concentrating capacity or the degree of progression of the disease.

## Conclusion

In the last few years, knowledge has accumulated on how uEVs should be separated and analyzed. In addition, it has become clear how they are related to composition in the renal tissues and how they are altered in disease states. uEVs have garnered significant attention as promising diagnostic and prognostic biomarkers in renal or urogenital disease. As an example of uEVs in clinical practice, ExoDx has reportedly led to an approximately 30% reduction in prostate biopsies [74]. As kidney biopsy is the established and highly informative method to directly analyze damaged tissues, it is hard to imagine uEV analysis replacing it. Instead, uEVs may be utilized as a new source of kidney tissue-derived information to supplement urinalysis and blood tests. Many diseases underlying childhood CKD are hard to detect using conventional urinalysis, and the advantages of non-invasive testing techniques are especially significant for children.

Although this review focuses on their diagnostic aspect, uEVs have recently been discussed for their active roles in renal physiology [80] or pathology [24], such as AKI to CKD transition through intracellular communication. uEVs even hold great potential for therapeutic applications such as kidney regeneration [23, 81]. Further advances on new diagnostic tools or therapies using EVs are anticipated to improve outcomes for childhood kidney diseases.
